# Long‐term cost of spouses’ informal support for dependent midlife stroke survivors

**DOI:** 10.1002/brb3.716

**Published:** 2017-05-03

**Authors:** Josefine Persson, Lars‐Åke Levin, Lukas Holmegaard, Petra Redfors, Mikael Svensson, Katarina Jood, Christina Jern, Christian Blomstrand, Gunilla Forsberg‐Wärleby

**Affiliations:** ^1^ Department of Clinical NeuroscienceInstitute of Neuroscience and Physiologythe Sahlgrenska Academy at University of GothenburgGothenburgSweden; ^2^ Health Metricsthe Sahlgrenska Academy at University of Gothenburg & Centre for Health Economics (CHEGU) at the University of GothenburgGothenburgSweden; ^3^ Department of Medical and Health ScienceLinköping UniversityLinköpingSweden; ^4^ Department of Clinical Pathology and GeneticsInstitute of Biomedicinethe Sahlgrenska Academy at University of GothenburgGothenburgSweden; ^5^ Stroke Centre Westthe Sahlgrenska Academy at University of GothenburgGothenburgSweden

**Keywords:** cost and cost analysis, informal support, opportunity cost, stroke, time costs, time‐diary

## Abstract

**Objectives:**

Stroke is a major global disease that requires extensive care and support from society and relatives. The aim of this study was to identify and quantify the long‐term informal support and to estimate the annual cost of informal support provided by spouses to their stroke surviving partner.

**Method:**

Data were based on the 7‐year follow‐up of the Sahlgrenska Academy Study on Ischemic Stroke. One‐third of the spouses stated that they provided support to their stroke surviving partner. The magnitude of the support was assessed with a study‐specific time‐diary and was estimated for independent and dependent stroke survivors based on the scores of the modified Rankin Scale. To deal with skewed data, a two‐part econometric model was used to estimate the annual cost of informal support.

**Result:**

Cohabitant dyads of 221 stroke survivors aged <70 at stroke onset were included in the study. Spouses of independent stroke survivors (*n* = 188) provided on average 0.15 hr/day of practical support and 0.48 hr/day of being available. Corresponding figures for spouses of dependent stroke survivors (*n* = 33) were 5.00 regarding practical support and 9.51 regarding being available. The mean annual cost of informal support provided for independent stroke survivors was estimated at €991 and €25,127 for dependent stroke survivor.

**Conclusion:**

The opportunity cost of informal support provided to dependent midlife stroke survivors is of a major magnitude many years after stroke onset and should be considered in economic evaluations of health care.

## 1 Introduction

Stroke is the foremost cause of adult disability (GBD, 2016). Stroke mortality has been declining for two decades, but the number of stroke survivors with disabilities is increasing (Feigin et al., 2014). Between 1990 and 2013, prevalent cases in adults aged 20–64 years increased significantly (Krishnamurthi et al., 2015). The survival rates are higher in the younger age groups (Rosengren et al., 2013), and thus, there are many relatives who are cohabitant with a stroke surviving family member in need of support for many years. A stroke in this age group often has a wide‐spread impact on the daily lives of the stroke survivors and their families (Grigorovich et al., 2015). Spouses of stroke survivors provide support and care for their partners to an extent far beyond what society can offer (Anderson, Linto, & Stewart‐Wynne, 1995; Claesson, Gosman‐Hedstrom, Johannesson, Fagerberg, & Blomstrand, 2000; Persson, Ferraz‐Nunes, & Karlberg, 2012). Stroke often leads to impaired physical, emotional, and cognitive abilities (Sudlow & Warlow, 1997), and therefore, the caregivers’ support covers a wide range of activities. Some of these activities are more task‐oriented, while others involve providing security by being available, often around the clock. Previous studies have estimated the average number of hours of care per day to be 4.6 after 6 months and 3.6 after 12 months poststroke (Tooth, McKenna, Barnett, Prescott, & Murphy, 2005). When surveillance was also included, the caregiving task was estimated to be 14.2 hr/day after 6 months (van Exel, Koopmanschap, van den Berg, Brouwer, & van den Bos, 2005). The estimated economic burden of informal caregiving per stroke survivor during the first year ranged from €3,100 to €7,600 (Fattore et al., 2012; Hickenbottom et al., 2002; Joo, Dunet, Fang, & Wang, 2014; Skolarus, Freedman, Feng, Wing, & Burke, 2016). However, the quantity or cost of informal support has only been estimated in a short‐term perspective and based on activities in daily life (ADL; Dewey et al., 2002; Hickenbottom et al., 2002; Joo et al., 2014; Skolarus et al., 2016; Tooth et al., 2005). Thus, knowledge is lacking concerning the long‐term costs and amount of informal support provided by spouses’ being available. Therefore, the objectives of this study were (1) to identify and quantify spouses’ informal support 7 years after stroke onset with a study‐specific time‐diary and (2) to estimate the annual cost of the informal support provided by spouses to their midlife stroke surviving partner.

## 2 Subjects and Methods

### 2.1 Subjects

Participants originated from the Sahlgrenska Academy Study on Ischemic Stroke, the design of which has been reported elsewhere (Jood, Ladenvall, Rosengren, Blomstrand, & Jern, 2005; Persson et al., 2015). In brief, 600 patients with ischemic stroke before the age of 70 were consecutively recruited from four stroke units within western Sweden during 1998–2003. Of these, 422 stroke survivors were cohabitant with their spouse and partner at baseline. In the 7‐year follow‐up, 299 stroke survivors were cohabitant, whereof 248 spouses were recruited to the study. The inclusion process and the analyses of drop‐outs of the total study population is previously described (Persson et al., 2015). One‐third (*n* = 80) of the spouses reported that they provided support to their partner. The data collection of informal support was made in a second step and of the 80 spouses providing support, 67 spouses were available and 53 fulfilled the data collection. Due to drop‐outs (*n* = 27), the total population in this study was 221. Thus, the study population consisted of 53 spouses who provided informal support and 168 spouses who provided no informal support. All responders gave informed consent and approved merging of data from different sources. The study was approved by the Regional Ethical Review Board in Gothenburg (reference number 413‐04, 622‐06, T715‐10).

### 2.2 Assessments and data collection

Questionnaires administered by a research nurse were used to collect socio‐demographic information about the stroke survivors and the spouses, as well as spouses’ support of housework tasks and contact with health care, and spouses’ perceptions concerning the length of time during which the stroke survivors could be left alone. The global disability of stroke survivors was assessed by the modified Rankin Scale (mRS; van Swieten, Koudstaal, Visser, Schouten, & van Gijn, 1988). To avoid that the subjective view of the interviewer might influence the results, the research nurse was trained to use key issues to distinguish different categories in a similar approach used in clinical trials (Harrison, McArthur, & Quinn, 2013).

**Figure 1:**
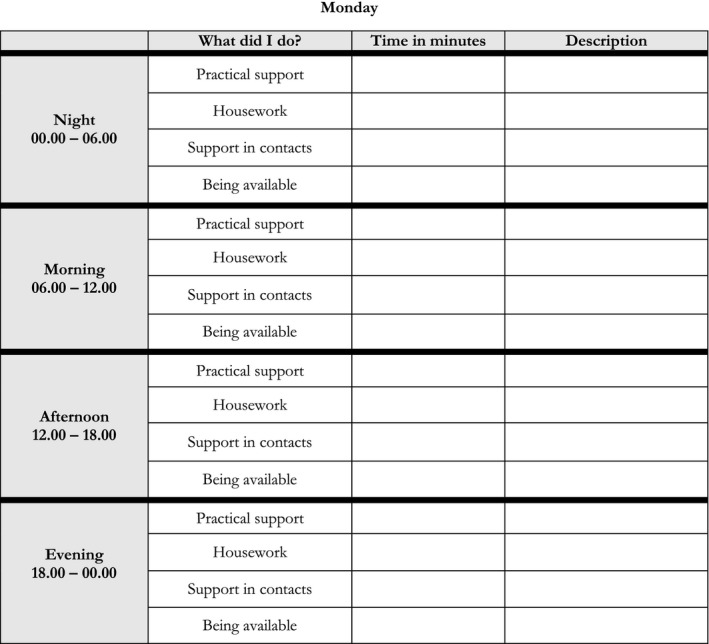
The time‐diary

Data concerning the quantity of informal support provided by the supporting spouses to their stroke surviving partner were collected with a specially designed time‐diary. First, the categories in the time‐diary were based on the suggested categories in the health economic literature such as; practical support including personal care, housework, and support in contact (van den Berg, Brouwer, & Koopmanschap, 2004). Second, a fourth category, “being available”, was included to capture an overall estimation of the spouse's time of support. This category was based on clinical experiences and previous research in which caregivers expressed a feeling of being bounded and unfree (Quinn, Murray, & Malone, 2014). Third, to facilitate the feasibility of the study, the time‐diary was divided in four blocks of time; night, morning, after‐noon, and evening and the spouse were asked to register the time spent in the different categories of support in each of the time‐periods. The time‐diary was illustrated in Figure 1.

Together with the time‐diary, a detailed information package on how to use the time‐diary and quantify the support was designed. Before the time‐diary was used in the study, it was discussed with people in various ages, testing the feasibility to complete the time‐diary and that the information package was easy to understand.

The time‐diary and the information package were sent to the spouses and they were asked to register their informal support during one for them normal week. The spouses were also asked to provide background information concerning occupational status and the amount of home care provided by the municipalities. Spouses were requested to record in the time‐diaries only “excess” time of informal support, that is, support that was specifically provided for the stroke survivor that the spouses did not provide prior to the stroke. In the analyses, the categories “Practical support,” “Housework,” and “Support in contacts”, were aggregated into one category, that is, “Practical support”. Further in the analyses, we just used a limit of 24 hr/day. Hence, if a spouse stated that he/she provided 24 hr of support by being available but also time of practical support, the time providing support by being available was reduced to match the limit of 24 hr/day.

### 2.3 Validation of the study‐specific time‐diary

The amount of time in each category in the time‐diary was compared with equivalent items from the questionnaire at the 7‐year follow‐up. Accordingly, the category “Practical support” was compared with questions regarding whether the spouses provided support with dressing, toileting, moving indoors and outdoors, and rehabilitation activities. The category “Housework” was compared with questions regarding whether the spouses had taken over some of the household chores that the stroke survivor had performed prior to the stroke, such as paying bills, writing letters, cleaning the home, and driving the car. The category “Support in contacts” was compared with whether the spouses provided any support in contacts with health care providers and other external authorities. The category “Being available” was compared with spouses’ reports concerning the length of time during which the stroke survivor could be alone without supervision.

### 2.4 Cost analysis

Informal support was estimated for both independent and dependent stroke survivors based on the mRS scores. A score of 0–2 indicated independence, while a score of 3–5 indicated dependence (Sulter, Steen, & De Keyser, 1999). The informal support was valued according to the opportunity cost method (Drummond, Sculpher, Torrance, O'Brien, & Stoddart, 2005), where the informal support is valued as the person's best alternative use of time, spent on work or leisure. The loss of production was valued by the human capital approach (Drummond et al., 2005) assuming that production loss is valued at market price, that is, gross salaries and payroll taxes. An hourly estimation including payroll taxes of €20 (exchange rate 0.10 from € to SEK; Statistics Sweden) was used for employed spouses. In the case of economically inactive spouses, that is, household workers, unemployed persons and pensioners, an hourly rate of €7, that is, 35% of the hourly loss of production of employed spouses was used as an estimation of their leisure time (Johannesson, Borgquist, Jönsson, & Råstam, 1991). In the cost analysis, joint production was considered for the category “Being available”, that is, when the spouses provided support to their partner that they themselves to some extent benefited from. Thus, the category “Being available” was in the base case valued at 50% of the leisure time, that is, an hourly rate of €3.5. Another proposed method to use is the replacement cost approach (Drummond et al., 2005), which estimates the cost of the informal support to the wage rate, including payroll taxes of a market substitute for the nonmarket good. Hence, for the category “Housework,” the cost is estimated to the hourly wage rate for housemaids, that is, €15 (Statistics Sweden). The categories “Practical support” and “Support in contact” are estimated to the hourly wage rate for nursing aids, that is, €16.4 (Statistics Sweden). The category “Being available,” is estimated to the hourly wage rate, for personal assistance, that is, €16.5 (Statistics Sweden).

To estimate the annual cost of spouses’ informal support, a 52‐week annual estimation was extrapolated from the weekly reported support in the time‐diaries. Four one‐way sensitivity analyses were performed (1) varying the unit cost of “Being available” between €1 and €6, (2) limiting the maximum possible time of support per day to 16 hr, (3) valuing the hourly rate of all the informal support when set at €20, that is, loss of gross salaries and payroll taxes, and (4) with the replacement cost approach.

### 2.5 Statistical analysis

The distribution of the variables was presented as mean and 95% confidence intervals (CIs) for continuous variables and as number and percentage for categorical variables. All significance tests were two‐sided and conducted at the 5% significance level. For comparison between groups, the nonparametric Mann–Whitney *U* test was used.

The two‐part model (tpm; Belotti, Deb, Manning, & Norton, 2015) is one approach to use when having a large numbers of zeroes in the data. The data in this study contained a large proportion of spouses who reported that they did not provide any informal support (*n* = 168), that is, true zeros, hence the tpm is an appropriate method to use to analyze the data. This approach has also been adopted in other studies estimating the cost of informal care (Hickenbottom et al., 2002). The tpm consists of two parts. Part I of the jointly estimated tpm was a binary choice model for estimating the probability of observing a positive outcome, that is, if spouses provided informal support. Part II was a regression model based on the observations with positive outcomes to estimate the association between hours/cost of informal support and the dependency/independency of the stroke survivors. The chosen approach was a logit for the first part and for the second part and an ordinary least squares with the natural logarithm of the outcome variable, that is, ln(hours) and ln(cost). For the retransformation from the ln‐scale to the raw cost scale, a Duan Smearing Approach was used (Duan, 1983).

The dependent variable was informal support, dichotomized as practical support and being available. The independent variable was mRS score, dichotomized as dependent (mRS 3–5) and independent (mRS 0–2). In Model 1, the dichotomized mRS scores were included as explanatory variables, while in Model 2, they were adjusted for spouses’ age, occupational status, and educational level. The results from Part I and Part II were combined to yield a predicted estimate of the hours per day and annual costs of practical support and being available for dependent and independent survivors, respectively. Due to a relatively small sample with skewed data, 95% CI for the mean hours per day and the mean annual cost was calculated using 1,000 percentile bootstrap replications.

The analyses were carried out using STATA statistical software (version 14; STATA, College Station, TX, USA), and the “tpm” command was used for the two‐part regression model, while the “margins” command was used for predictions.

## 3 Results

The mean (95% CI) ages of the spouses and stroke survivors were 62 (61–63) and 63 (61–65), respectively, and 66% and 34% were females, respectively. As judged by mRS data at the 7‐year follow‐up, 15% were spouses of dependent (mRS ≥ 3) stroke survivors, and 85% were spouses of independent (mRS ≤ 2) stroke survivors. The spouses of dependent stroke survivors were older (*p *=* *.003), and more of them were retired (*p *=* *.021) compared to spouses of the independent stroke survivors. The gender distribution did not differ between the two spouse groups. The dependent stroke survivors were older (*p *=* *.004) and received more hours of formal care per day (*p *=* *.012) compared to the independent stroke survivors. At the 7‐year follow‐up, 8 (24%) of the dependent stroke survivors and 23 (12%) of the independent stroke survivors had had a recurrent stroke. The demographic features of the study population are shown in Table 1.

**Table 1:** Demographic features of the study population

	Spouses of independent stroke survivors (%) *n* = 188	Spouses of dependent stroke survivors (%) *n* = 33	Independent stroke survivors (%) *n* = 188	Dependent stroke survivors (%) *n* = 33
Mean age, years (95% CI)	61 (60–63)	66 (63–69)	62 (61–64)	66 (64–71)
Female sex	124 (66)	21 (64)	63 (34)	12 (36)
Education				
Secondary or less	66 (35)	20 (61)	65 (34)	12 (36)
High school	64 (34)	5 (15)	69 (37)	12 (36)
University	58 (31)	8 (24)	54 (29)	8 (24)
Occupation^a^				
Employed	90 (48)	8 (24)	63 (34)	0 (0)
Retired	85 (45)	22 (67)	104 (55)	24 (73)
Unemployed, sick leave, other	18 (10)	3 (9)	45 (24)	7 (21)
Housing and support				
Living at home			188 (100)	24 (73)
Living at home with home aid			1 (0.5)	6 (18)
Living at home with personal assistant			0 (0)	3 (9)
Living at nursing home			0 (0)	1 (3)
Hours of formal home care per day (95% CI)			0.01 (0–0.02)	0.71 (0.10–1.32)
Support received from spouses^b^			27 (14)	31 (94)
Support provided by spouses^c^	22 (12)	31 (94)		
Children <18 living at home	25 (13)	2 (6)		

CI, confidence interval.

a Sum not equal to 100% due to multiple answering alternatives.

b Reported by the stroke survivors in the 7‐year questionnaire.

c Reported by the spouses in the 7‐year questionnaire.

The stroke survivors who were lost between baseline and the 7‐year follow‐up had poorer global disability, measured with the mRS (*p *<* *.001) at 3 months after stroke onset. More male than female stroke survivors were lost to follow‐up, but they did not differ in age.

The recruited spouses (*n* = 53) in the time‐diary study did not differ concerning age, sex, occupational status, level of education, nor the global disability of the included stroke survivors compared to the drop‐outs (*n* = 27). Of the drop‐outs, 13 were not available due to either the stroke survivor or the spouse had deceased during the time between the questionnaire and the time‐diary study. The spouses that received the time‐diary but did not complete it (*n* = 14), the mean age was 66 years (range: 50–81 years). The occupational status of these drop‐outs was 33% employed, 53% retired, and 13% unemployed or on sick leave. The results from the validation of the time‐diary showed that the informal support reported in the time‐diary were consistent with the support reported in the questionnaires (Table 2).

**Table 2:** Comparison of informal support reported in the time‐diaries with informal support reported in the questionnaires

Questionnaire in the 7‐year follow‐up of SAHLSIS	Mean (95% CI) hours of informal support per day in each category reported in the time‐diary			
	Practical support	Housework	Support in contacts	Being available
Reported support	0.91 (0.60–1.21)	1.62 (1.17–2.07)	0.78 (0.43–1.13)	
Reported no support	—	0.53 (0–1.29)	0.51 (0.21–0.81)	
Able to be alone less than half a day				5.95 (1.98–9.93)
Able to be alone more than half a day				3.42 (1.61–5.23)

SAHLSIS, Sahlgrenska Academy Study on Ischemic Stroke; CI, confidence interval.

**Figure 2:**
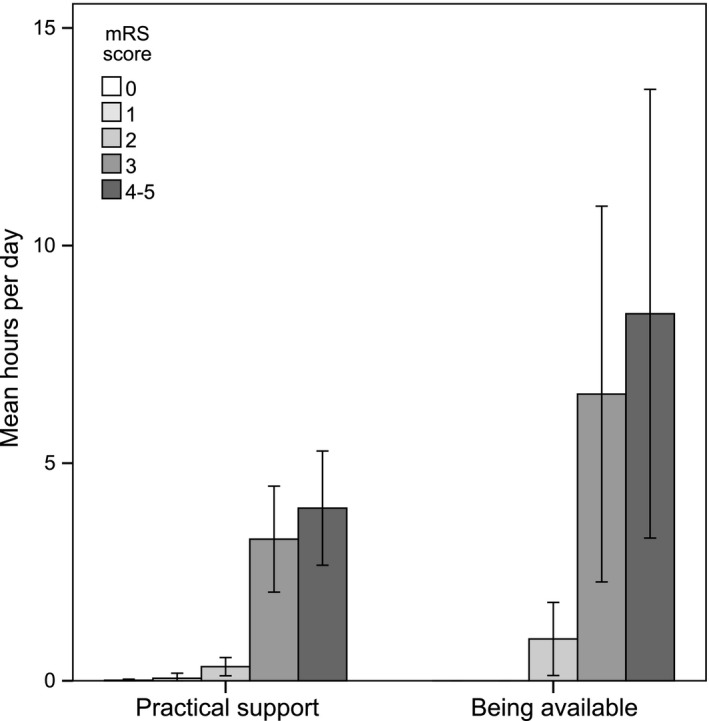
Practical support and being available in hours per day (95% confidence interval), by modified Rankin Scale (mRS) score

As illustrated by Figure 2, spouses of stroke survivors with mRS score 3–5 provided informal support to a greater extent, compared to spouses of stroke survivors with mRS score 0–2. The results from the time‐diaries show that the spouses provided support around the clock and gave the majority of their informal care within the category “Being available” (Fig. S1). There were no statistical differences regarding age, sex, occupational status, or level of education concerning the quantity of provided support.

The estimated hours per day of support provided by spouses of independent and dependent stroke survivors were 0.63 and 14.51 hr/day, respectively. The estimated annual cost of informal support provided for dependent stroke survivors was €25,127 and for independent stroke survivor €991 (Table 3). The full output from the tpms is available in the Tables S1 and S2.

**Table 3:** Predicted hours per day and annual cost of practical support and being available based on the two‐part model

	Independent stroke survivors	Dependent stroke survivors
Two‐part model 1		
Hours of practical support per day	0.15 (0.02–0.28)**	4.38 (2.94–5.82)***
Hours of being available per day	0.46 (−0.07 to 1.01)*	9.66 (2.88–16.44)***
Total hours per day	0.61 (0.02–0.92)**	14.04 (4.86–17.94)***
Annual cost for practical support	406 (64–749)**	11,884 (7,982–15,787)***
Annual cost for being available	568 (−92 to 1,227)*	11,714 (3,486–19,942)***
Total annual cost	974 (122–1,753)**	23,598 (14,409–36,070)***
Two‐part model 2		
Hours of practical support per day	0.15 (0.01–0.30)**	5.00 (2.76–7.24)***
Hours of being available per day	0.48 (−0.14 to 1.09)	9.51 (1.35–17.68)***
Total hours per day	0.63 (0.03–0.98)*	14.51 (3.36–18.76)***
Annual cost for practical support	412 (−14.4 to 838)*	13,539 (7,030–20,049)***
Annual cost for being available	579 (−200 to 1,358)*	11,588 (1,897–21,278)***
Total annual cost	991 (8.43–1,893)**	25,127 (13,629–39,991)***

Costs are presented in € (2015). Two‐part model 2: adjusted for spouses’ sex and occupational status.

Level of significance: ***1%, **5%, *10%.

To analyze the robustness of the results, four one‐way sensitivity analyses were employed and presented in Table 4. The results were most sensitive to how the support was valued, that is, if it was valued as loss of gross salaries and payroll taxes instead of loss of leisure time.

**Table 4:** Sensitivity analyses

	Independent stroke survivors	Dependent stroke survivors
Being available, valued at €1	652 (32–1,273)**	18,156 (9,830–26,483)***
Being available, valued at €6	1,219 (2–2,441)**	35,871 (15,183–56,558)***
Limit of 16 support hours per day	819 (51–1,589)**	23,788 (11,633–35,952)***
Informal support valued at €20	1,862 (50–3,773)**	41,164 (15,578–66,751)***
Replacement cost approach	1,720 (61–3,378)**	57,215 (31,002–83,428)***

Total annual costs are presented in € (2015). 95% confidence interval estimated with percentile bootstrap with 1,000 replications in parentheses.

Costs were adjusted for spouses’ sex and occupational status.

Level of significance: ***1%, **5%, *10%.

## 4 Discussion

This study aimed to identify and quantify the amount of support provided by the spouses to their midlife stroke surviving partner 7 years after stroke onset and to estimate the annual long‐term cost of the spouses’ informal support. To achieve this aim, we used a study‐specific time‐diary including categories previously recommended in an overview of methods and applications when estimating the cost of informal care (van den Berg et al., 2004). In addition, we also included another category, that is, “Being available,” to capture an overall estimation of the informal support. A study‐specific time‐diary was designed for this study since no previous time‐diaries in the literature were appropriate for this study. When designing the time‐diary, we weighed a time‐diary with more categories and shorter time‐slots against a time‐diary more feasible to complete. In comparison to a previously suggested time‐diary to value the informal support into monetary term (van den Berg & Spauwen, 2006), the time‐diary used in this study was in a simpler form concerning both number of categories and the number of daily time‐slots to facilitate the feasibility of the study. A disadvantage with our approach might be that we did not schedule the time‐diary so that responders could report only one activity at the same time, to avoid joint production, but on the other hand, strict scheduling might have introduced increased difficulty for the spouse to judge. This was mainly a problem in the category “Being available” where 25% of the spouses in the time‐diary study indicated that they provided support for 20–24 hr/day. Hence, we made an assumption to value the support by being available to 50% being joint. Further, the spouses reported their informal support during 1 week while previous studies using a time‐diary method have collected data during different periods of time, such as 2 days (van den Berg & Spauwen, 2006) and during 4 weeks (Flyckt, Lothman, Jorgensen, Rylander, & Koernig, 2013). Further, according to a recent systematic review of the valuation of informal support (Oliva‐Moreno, Trapero‐Bertran, Pena‐Longobardo, & Del Pozo‐Rubio, 2017), none of the included articles used a time‐diary as a method to measure time and cost of informal care. We thought it was of value to estimate the time of informal support with a time‐diary.

The rationale for highlighting the informal support provided by spouses was twofold. Firstly, spouses of midlife stroke survivors often also have responsibilities for the family situation, economy, and a professional life (Green & King, 2007). Thus, spouses of younger stroke survivors may experience a greater conflict between their regular daily family and household chores, working lives, and the support provided to their partner, in comparison to other caregivers such as children and friends. Secondly, given that younger stroke survivors have longer survival time, in line with the secular trend of decreasing risk of mortality (Rosengren et al., 2013), spouses must provide support to their partner over a longer period of time compared to older stroke survivors.

In previous studies, the informal support estimation was based on ADL and instrumental ADL (Dewey et al., 2002; Hickenbottom et al., 2002; Joo et al., 2014; Skolarus et al., 2016; Tooth et al., 2005). However, according to our study, these activities represent only a minor proportion of the informal support provided by the spouses of stroke survivors. The majority of the reported informal support in this study consisted of time being available.

Further knowledge of the cost of informal support is important in any comprehensive estimation of the societal economic burden for a disease and when evaluating the cost‐effectiveness of a health care intervention. Our study estimated that the annual cost of informal support for dependent stroke survivors exceeded previous estimates (Fattore et al., 2012; Hickenbottom et al., 2002; Joo et al., 2014; Skolarus et al., 2016). The discrepancy between our results and those from previous studies is mainly due to our effort to capture an overall picture of the informal support, including costs of both practical support and support by being available. A further reason may be that our study only included spouses of stroke survivors compared to previous studies including family, friends, and voluntary personnel. Thus, the daily average of hours of informal support may be higher among spouses. Cohabitant spouses might provide informal support that otherwise would have been provided by formal care, such as home care to stroke survivors living alone. The previous cost estimates used a limit of 16 support hours per day, that is, excluding the support provided during the night. However, in our estimations, we did not use a limited number of hours per day, as spouses reported providing support 24 hr/day, that is, also during the night. Furthermore, compared to previous studies, we reported informal support for independent and dependent stroke survivors based on the mRS score and were thereby able to examine the support provided, according to dependency level. Hickenbottom et al. (Hickenbottom et al., 2002) estimated the quantity of informal support based on stroke survivors ≥70 years at stroke onset, with and without self‐related health problems (SRHP). Their study showed that stroke survivors with SRHP had both an increased likelihood of receiving informal support from informal caregivers and more average weekly caregiving hours. In contrast with Joo et al. (2014), we did not collect data on informal support prior to the stroke onset, although we asked the spouses to report solely the excess time of support due to the stroke. In contrast to Fattore et al. (2012), Joo et al. (2014), and Skolarus et al. (2016), we used the opportunity cost method to estimate the economic value of the informal support. This method was chosen due to the fact that the replacement method estimates the economic value of the informal support as if a professional health carer provided the same care, which can lead to an overvaluation (Drummond et al., 2005). The results in our sensitivity analysis showed that the result were sensitive to the chosen method of valuing informal support, as expected. The result changed substantially when the support was valued as loss of gross salaries with payroll taxes, and with the replacement cost approach, in comparison of loss of leisure time.

In the validation of the time‐diary, the questions regarding practical support, housework, and support in contacts in the questionnaire were comparable to each category in the time‐diary. However, in the questionnaire, the spouses were asked to report the length of time during which the stroke survivors could be alone without supervision, but in the time‐diary the spouses were asked to report the length of time during which they were available. Thus, there might be a discrepancy between these measures and further research concerning supporting spouses is necessary to understand the mechanisms underlying the perceived need of being available.

The main advantage of our study is the well‐described study population, with high response rate, used to delineate the informal support provided by spouses of stroke survivors. Furthermore, we used a study‐specific diary method that minimized the risk of recall bias (van den Berg et al., 2004), compared to previous studies relying on the recall method, that is, questionnaires or interviews with either the stroke survivor or the relatives. However, the study has some limitations. Firstly, we had longitudinal data solely for the stroke survivors, and not for the spouses. Since a larger proportion of stroke survivors with higher mRS scores at 3 months were lost to follow‐up at 7 years (Persson et al., 2015), the amount of informal support provided and by how many of the spouses can be an underestimation. Further studies are needed to investigate spouses’ informal support within a longitudinal perspective. Secondly, the sample that reported their support in the time‐diary is small, which limits the generalizability of the results due to possible type II error. Nevertheless, previous studies have also shown that the severity of the stroke is indicative of the amount of comprehensive informal support needed. Thirdly, the diary used was study specific and thus has not been validated other than with comparisons with the 7‐year questionnaire. Fourthly, we did not ask questions about the spouse's income and instead used the average income in western Sweden from Statistics Sweden when valuing the informal support with the opportunity cost method. Further studies are necessary to study if the income level might have had an impact on spouses’ willingness to reduce work time to provide support.

## 5 Conclusion

Stroke is a complex disease, affecting each individual differently and thereby also the spouses. However, our results show that the informal support provided by the spouses is associated with the dependency of the stroke survivors. Consequently, the opportunity cost of informal support provided to dependent young and mid‐aged stroke survivors is of a major magnitude many years after stroke onset and should be considered in economic evaluations of health care.

## Conflict of Interest

The authors declare no conflicts of interest.

## References

[brb3716-bib-0001] Anderson, C. S. , Linto, J. , & Stewart‐Wynne, E. G. (1995). A population‐based assessment of the impact and burden of caregiving for long‐term stroke survivors. Stroke, 26, 843–849. 7740578 10.1161/01.str.26.5.843

[brb3716-bib-0002] Belotti, F. , Deb, P. , Manning, W. , & Norton, E. (2015). Twopm: Two‐part models. Stata Journal, 15, 3–20.

[brb3716-bib-0003] Briggs, A. (2001). Uncertainty in economic evaluation and presenting the results In Drummond M. (Ed.), Economic evaluation in health care: Merging theory with practice. Oxford, UK: Oxford University Press.

[brb3716-bib-0004] Claesson, L. , Gosman‐Hedstrom, G. , Johannesson, M. , Fagerberg, B. , & Blomstrand, C. (2000). Resource utilization and costs of stroke unit care integrated in a care continuum: A 1‐year controlled, prospective, randomized study in elderly patients: The Goteborg 70 + stroke study. Stroke, 31, 2569–2577. 11062277 10.1161/01.str.31.11.2569

[brb3716-bib-0005] Dewey, H. M. , Thrift, A. G. , Mihalopoulos, C. , Carter, R. , Macdonell, R. A. L. , McNeil, J. J. , & Donnan, G. A. (2002). Informal care for stroke survivors: Results from the North East Melbourne Stroke Incidence Study (NEMESIS). Stroke, 33, 1028–1033. 11935056 10.1161/01.str.0000013067.24300.b0

[brb3716-bib-0006] Drummond, F. , Sculpher, M. , Torrance, G. , O'Brien, B. , & Stoddart, G. (2005). Methods for the economic evaluation of health care programmes (3rd ed.). Oxford, UK: Oxford University Press.

[brb3716-bib-0007] Duan, N. (1983). Smearing estimate: A nonparametric retransformation method. Journal of the American Statistical Association, 78, 605–610.

[brb3716-bib-0008] Fattore, G. , Torbica, A. , Susi, A. , Giovanni, A. , Benelli, G. , Gozzo, M. , & Toso, V. (2012). The social and economic burden of stroke survivors in Italy: A prospective, incidence‐based, multi‐centre cost of illness study. BMC Neurology, 12, 137. 23150894 10.1186/1471-2377-12-137 PMC3536660

[brb3716-bib-0009] Feigin, V. L. , Forouzanfar, M. H. , Krishnamurthi, R. , Mensah, G. A. , Connor, M. , Bennett, D. A. , … Global Burden of Diseases, Injuries, and Risk Factors Study 2010 (GBD 2010) and the GBD Stroke Experts Group . (2014). Global and regional burden of stroke during 1990‐2010: Findings from the Global Burden of Disease Study 2010. The Lancet, 383, 245–254. 10.1016/s0140-6736(13)61953-4 PMC4181600 24449944

[brb3716-bib-0010] Flyckt, L. , Lothman, A. , Jorgensen, L. , Rylander, A. , & Koernig, T. (2013). Burden of informal care giving to patients with psychoses: A descriptive and methodological study. International Journal of Social Psychiatry, 59, 137–146. 22100570 10.1177/0020764011427239 PMC3652598

[brb3716-bib-0011] GBD (2016). Global, regional, and national disability‐adjusted life‐years (DALYs) for 315 diseases and injuries and healthy life expectancy (HALE), 1990‐2015: A systematic analysis for the Global Burden of Disease Study 2015. The Lancet, 388, 1603–1658. 10.1016/S0140-6736(16)31460-X PMC5388857 27733283

[brb3716-bib-0012] Green, T. L. , & King, K. M. (2007). The trajectory of minor stroke recovery for men and their female spousal caregivers: Literature review. Journal of Advance Nursing, 58, 517–531. 10.1111/j.1365-2648.2007.04321.x 17542802

[brb3716-bib-0013] Grigorovich, A. , Forde, S. , Levinson, D. , Bastawrous, M. , Cheung, A. M. , & Cameron, J. I. (2015). Restricted participation in stroke caregivers: Who is at risk? Archives of Physical Medicine and Rehabilitation, 96, 1284–1290. 25819264 10.1016/j.apmr.2015.03.004

[brb3716-bib-0015] Harrison, J. , McArthur, K. , & Quinn, T. (2013). Assessment scales in stroke: Clinimetric and clinical considerations. Journal of Clinical Interventions in Aging, 8, 201–211. 23440256 10.2147/CIA.S32405 PMC3578502

[brb3716-bib-0016] Hickenbottom, S. L. , Fendrick, A. M. , Kutcher, J. S. , Kabeto, M. U. , Katz, S. J. , & Langa, K. M. (2002). A national study of the quantity and cost of informal caregiving for the elderly with stroke. Neurology, 58, 1754–1759. 12084872 10.1212/wnl.58.12.1754

[brb3716-bib-0017] Johannesson, M. , Borgquist, L. , Jönsson, B. , & Råstam, L. (1991). The cost of treating hypertension—An analysis of different cut‐off points. Health Policy, 18, 141–150. 10112585 10.1016/0168-8510(91)90095-f

[brb3716-bib-0018] Joo, H. , Dunet, D. , Fang, J. , & Wang, G. (2014). Cost of informal caregiving associated with stroke among the elderly in the United States. Neurology, 83, 1831–1837. 25305152 10.1212/WNL.0000000000000986 PMC4365923

[brb3716-bib-0019] Jood, K. , Ladenvall, C. , Rosengren, A. , Blomstrand, C. , & Jern, C. (2005). Family history in ischemic stroke before 70 years of age: The Sahlgrenska Academy Study on Ischemic Stroke. Stroke, 36, 1383–1387. 15933254 10.1161/01.STR.0000169944.46025.09

[brb3716-bib-0020] Krishnamurthi, R. V. , Moran, A. E. , Feigin, V. L. , Barker‐Collo, S. , Norrving, B. , Mensah, G. A. , … GBD 2013 Stroke Panel Experts Group . (2015). Stroke prevalence, mortality and disability‐adjusted life years in adults aged 20‐64 years in 1990‐2013: Data from the global burden of disease 2013 study. Neuroepidemiology, 45, 190–202. 26505983 10.1159/000441098

[brb3716-bib-0021] Oliva‐Moreno, J. , Trapero‐Bertran, M. , Pena‐Longobardo, L. M. , & Del Pozo‐Rubio, R. (2017). The valuation of informal care in cost‐of‐illness studies: A systematic review. Pharmacoeconomics, 35, 331–345. 27848219 10.1007/s40273-016-0468-y

[brb3716-bib-0022] Persson, J. , Ferraz‐Nunes, J. , & Karlberg, I. (2012). Economic burden of stroke in a large county in Sweden. BMC Health Services Research, 12, 341. 23013284 10.1186/1472-6963-12-341 PMC3506527

[brb3716-bib-0023] Persson, J. , Holmegaard, L. , Karlberg, I. , Redfors, P. , Jood, K. , Jern, C. , … Forsberg‐Wärleby, G. (2015). Spouses of stroke survivors report reduced health‐related quality of life even in long‐term follow‐up: Results from Sahlgrenska Academy Study on Ischemic Stroke. Stroke, 46, 2584–2590. 26294675 10.1161/STROKEAHA.115.009791

[brb3716-bib-0024] Quinn, K. , Murray, C. , & Malone, C. (2014). Spousal experiences of coping with and adapting to caregiving for a partner who has a stroke: A meta‐synthesis of qualitative research. Disability and Rehabilitation, 36, 185–198. 23597001 10.3109/09638288.2013.783630

[brb3716-bib-0025] Rosengren, A. , Giang, K. W. , Lappas, G. , Jern, C. , Toren, K. , & Bjorck, L. (2013). Twenty‐four‐year trends in the incidence of ischemic stroke in Sweden from 1987 to 2010. Stroke, 44, 2388–2393. 23839506 10.1161/STROKEAHA.113.001170

[brb3716-bib-0026] Skolarus, L. , Freedman, V. , Feng, C. , Wing, J. , & Burke, J. (2016). Care recieved by elderly US stroke survivors may be underestimated. Stroke, 47, 2090–2095. 27387990 10.1161/STROKEAHA.116.012704 PMC4961527

[brb3716-bib-0027] Statistics Sweden . (2016). Average basic salary, monthly salary and women′s salary as a percentage of men′s salary by region, sector, occupation and sex. Retrieved from http://www.scb.se/

[brb3716-bib-0028] Sudlow, C. L. , & Warlow, C. P. (1997). Comparable studies of the incidence of stroke and its pathological types: Results from an international collaboration. International Stroke Incidence Collaboration. Stroke, 28, 491–499. 9056601 10.1161/01.str.28.3.491

[brb3716-bib-0029] Sulter, G. , Steen, C. , & De Keyser, J. (1999). Use of the Barthel index and modified Rankin scale in acute stroke trials. Stroke, 30, 1538–1541. 10436097 10.1161/01.str.30.8.1538

[brb3716-bib-0030] Tooth, L. , McKenna, K. , Barnett, A. , Prescott, C. , & Murphy, S. (2005). Caregiver burden, time spent caring and health status in the first 12 months following stroke. Brain Injury, 19, 963–974. 16263639 10.1080/02699050500110785

[brb3716-bib-0031] van den Berg, B. , Brouwer, W. B. , & Koopmanschap, M. A. (2004). Economic valuation of informal care. An overview of methods and applications. The European Journal of Health Economics, 5, 36–45. 15452763 10.1007/s10198-003-0189-y

[brb3716-bib-0032] van den Berg, B. , & Spauwen, P. (2006). Measurement of informal care: An empirical study into the valid measurement of time spent on informal caregiving. Health Economics, 15, 447–460. 16389664 10.1002/hec.1075

[brb3716-bib-0033] van Exel, N. J. , Koopmanschap, M. A. , van den Berg, B. , Brouwer, W. B. , & van den Bos, G. A. (2005). Burden of informal caregiving for stroke patients. Identification of caregivers at risk of adverse health effects. Cerebrovascular Diseases, 19, 11–17. 15528879 10.1159/000081906

[brb3716-bib-0034] van Swieten, J. C. , Koudstaal, P. J. , Visser, M. C. , Schouten, H. J. , & van Gijn, J. (1988). Interobserver agreement for the assessment of handicap in stroke patients. Stroke, 19, 604–607. 3363593 10.1161/01.str.19.5.604

