# Supplementary figures and images for: Long‐term cost of spouses’ informal support for dependent midlife stroke survivors
Source: Brain Behav. 2017 May 3;7(6):e00716. doi: 10.1002/brb3.716 (PMC5474719; doi:10.1002/brb3.716)

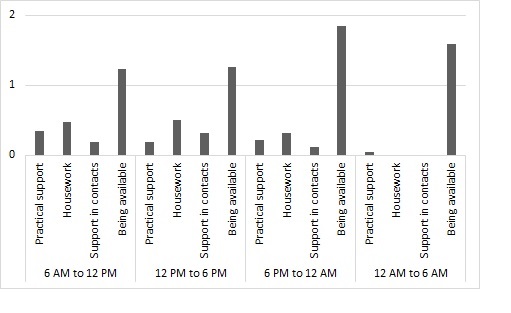

Supplement: Supplementary file 1 [file BRB3-7-e00716-s001.jpg]
