# Supplementary material for: Long‐term cost of spouses’ informal support for dependent midlife stroke survivors
Source: Brain Behav. 2017 May 3;7(6):e00716. doi: 10.1002/brb3.716 (PMC5474719; doi:10.1002/brb3.716)
Supplement: Supplementary file 2 [file BRB3-7-e00716-s002.docx]

**Supplemental Table 1.** Results from the two-part model regarding hours of informal support per day.

|  | Two-part model 1 | | Two-part model 2 | |
| --- | --- | --- | --- | --- |
|  | First part | Second part | First part | Second part |
| Practical support |  |  |  |  |
| Dependent^1^ | 4.29 (0.60)*** | 1.10 (0.01)** | 4.36 (0.85)*** | 1.21 (0.44)** |
| Male^2^ |  |  | -0.59 (0.81) | -0.02 (0.34) |
| Retired^3^ |  |  | 0.24 (0.61) | -0.46 (0.45) |
| Other^3^ |  |  | 0.07 (0.73) | -0.62 (0.58) |
| Intercept | -2.31 (0.26)*** | 0.10 (0.362)*** | -2.13 (0.48)*** | 0.44 (0.42)*** |
| Observations | 221 | 46 | 221 | 46 |
|  |  |  |  |  |
| Being available |  |  |  |  |
| Dependent^1^ | 3.34 (0.50)*** | 0.64 (0.60) | 3.29 (0.59)*** | 0.65 (0.70) |
| Male^2^ |  |  | 0.13 (0.55) | -0.21 (0.72) |
| Retired^3^ |  |  | 0.22 (0.67) | 0.02 (0.84) |
| Other^3^ |  |  | -0.12 (0.82) | -0.68 (0.99) |
| Intercept | -2.78 (0.33)*** | 1.16 (0.50)** | -2.90 (0.63)*** | 1.35 (0.74) |
| Observations | 221 | 33 | 221 | 33 |

Standard errors estimated with percentile bootstrap with 1000 replications in parentheses. First part was a logit and the second part and an ordinary least squares (OLS) with the natural logarithm of the outcome variable, i.e. ln(hours).

Level of significance: ***1%, **5%, *10%.

Model 2: adjusted for spouses’ sex and occupational status (other equals part time employment, part time retirement, unemployment, sick leave and being a student).

Following references are used:

^1^Independent stroke survivor

^2^Females

^3^Employed

**Supplemental Table 2.** Results from the two-part model regarding annual cost of informal support. Costs are presented in € (2015).

|  | Two-part model 1 | | Two-part model 2 | |
| --- | --- | --- | --- | --- |
|  | First part | Second part | First part | Second part |
| Practical support |  |  |  |  |
| Dependency^1^ | 4.29 (0.60)*** | 1.10 (0.28)** | 4.36 (0.85)** | 1.21 (0.44)** |
| Males^2^ |  |  | -0.59 (0.81) | -0.02 (0.32) |
| Retired^3^ |  |  | 0.24 (0.61) | -0.46 (0.44) |
| Other^3^ |  |  | 0.07 (0.73) | -0.63 (0.54) |
| Intercept | -2.31 (0.26)*** | 8.00 (0.27)*** | -2.13 (0.48)*** | 8.35 (0.411)*** |
| Observations | 221 | 46 | 221 | 46 |
|  |  |  |  |  |
| Being available |  |  |  |  |
| Dependency^1^ | 3.34 (0.50)*** | 0.64 (0.59) | 3.29 (0.59)** | 0.65 (0.70) |
| Males^2^ |  |  | 0.13 (0.55) | -0.21 (0.71) |
| Retired^3^ |  |  | 0.22 (0.67) | 0.02 (0.81) |
| Other^3^ |  |  | -0.12 (0.82) | -0.68 (0.98) |
| Intercept | -2.78 (0.33)*** | 8.27 (0.49) | -2.90 (0.63) | 8.57 (0.75)*** |
| Observations | 221 | 33 | 221 | 33 |

Standard errors estimated with percentile bootstrap with 1000 replications in parentheses. First part was a logit and the second part and an ordinary least squares (OLS) with the natural logarithm of the outcome variable, i.e. ln(cost).

Level of significance: ***1%, **5%, *10%.

Model 2: adjusted for spouses’ age and occupational status (other equals part time employment, part time retirement, unemployment, sick leave and being a student).

Following references are used:

^1^Independent stroke survivor

^2^Females

^3^Employed

Figure S1. Reported mean informal support in hours per day in each category in the time-diaries (n=53).
